# The complete chloroplast genome of *Arachis lutescens* Krapov. & Rigoni (Fabaceae)

**DOI:** 10.1080/23802359.2024.2353230

**Published:** 2024-05-31

**Authors:** Hexin Cui, He Xu, Yu Zhang, Chunrui Xu, Han Wang, Qinghua Li

**Affiliations:** a School of Nursing and Health, Zhengzhou University, Zhengzhou, China; b School of Life Sciences, Zhengzhou University, Zhengzhou, China; c College of Life Sciences, Henan Agricultural University, Zhengzhou, China

**Keywords:** Chloroplast genome, phylogenetic analysis, *Arachis lutescens*, Fabaceae

## Abstract

*Arachis lutescens* Krapov. & Rigoni 1958 is an important species due to their potentially extensive applications for cultivated peanut breeding. The whole chloroplast genome of *A. lutescens* was successfully assembled and annotated for the first time. The complete chloroplast genome of *A. lutescens* is a typically circular structure of 156,398 bp with a GC content of 36.3%. It comprises a large single-copy (LSC) region of 85,950 bp, a small single-copy (SSC) region of 18,800 bp, and two inverted repeat regions (IRs) of 25,824 bp, each. The plastome of *A. lutescens* contains a total of 125 genes, including 81 protein-coding genes, 36 tRNAs, and eight rRNAs. The phylogenetic analysis strongly supports the close relationship between *A. lutescens* and cultivated peanut clades. This study contributes to our understanding of the molecular characteristics and evolutionary relationships of this plant species.

## Introduction

**Figure 1.**
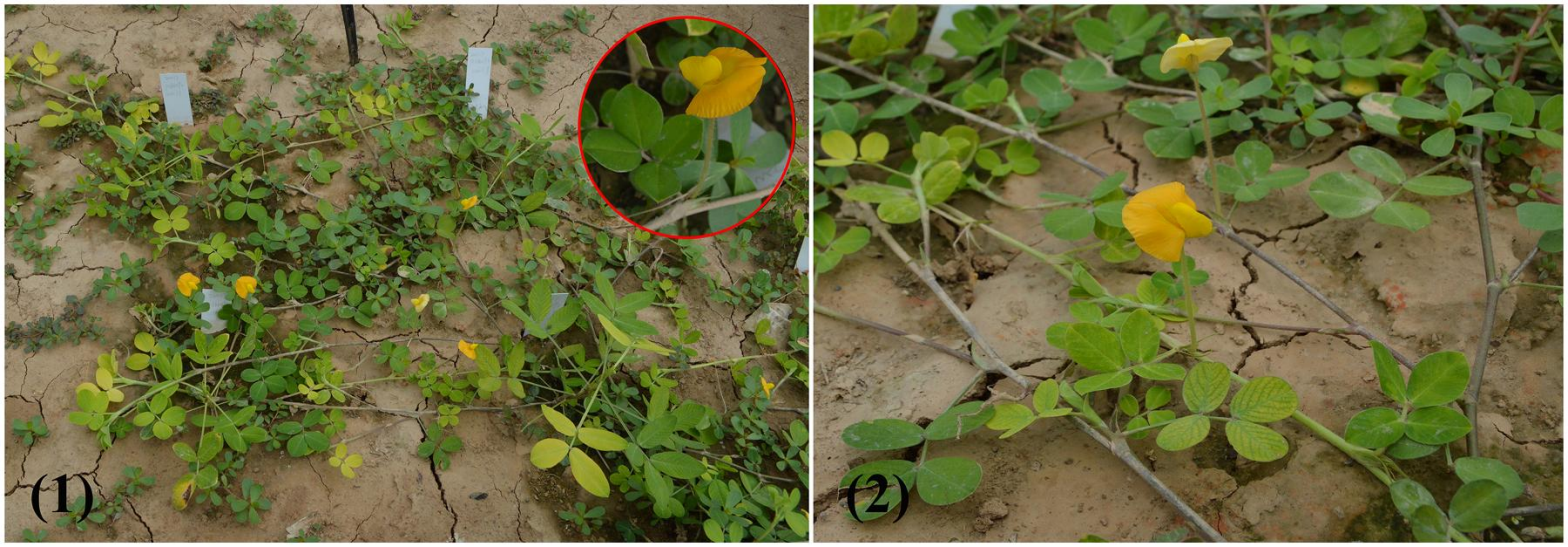
Natural ecological environment in the *Arachis lutescens* Krapov. & Rigoni planting area (1). Morphological characteristics of *A. lutescens*. Species photos were taken by the author in Henan Academy of Agricultural Sciences, Zhengzhou, Henan province, without any copyright issues.

The genus Arachis comprises 81 perennial or annual herb species, and was classified into nine described sections, namely sect. *Arachis*, sect. *Caulorrhizae*, sect. *Erectoides*, sect. *Extranervosae*, sect. *Heteranthae*, sect. *Procumbentes*, sect. *Rhizomatosae*, sect. *Trierectoides*, and sect. *Triseminatae* (Krapovickas et al. 2007; Bertioli et al. 2011; Stalker 2017). As an economically important cultivated oilseed crop species, *A. hypogaea* L., this genus has gained significant research interest (Bertioli et al. 2011; Yin et al. 2018; Bertioli et al. 2019; Zhuang et al. 2019). *Arachis lutescens* Krapov. & Rigoni is known as a wild perennial *Arachis* species, belongs to sect. *Extranervosae*, with prefers to grow in soils consisting of angular gravel (Jarvis et al. 2003). It is widely distributed in the south of 14°30′S, where flooded places or places subject to flooding (Krapovickas et al. 2007). This species is morphologically similar to *A. prostrata* Benth., as plants prostrate, leaflets without margin marked, and small fruits **(**Figure 1**)**, but differs from it in pegs have no adventitious roots and leaflets are oblong to obovate-ovate (Krapovickas et al. 2007). In addition, *A. lutescens* has high crude protein content, strong barren, and trampling resistance, which was a potential high-quality wild resource for peanut breeding. The genomic data of chloroplast genomes for various species of *Arachis* have been published, which have contributed to examining the phylogenetic relationships of *Arachis* (Yin et al. 2017; Wang et al. 2018, 2019; Tian et al. 2021; Zhang et al. 2021). The genus *Arachis* has about 27 wild peanut species; no species of the sect. *Extranervosae* has been reported. Therefore, it is necessary to investigate molecular data to distinguish the phylogenetic relationship between *A. lutescens* and peanut in the genus *Arachis*. Chloroplast plays a vital role in the evolution of all plant species. The chloroplast genomes of land plants are relatively conserved in structure, GC content, gene number, and gene arrangement among angiosperms (Xu et al. 2015). These characteristics of chloroplast genome allow for its wide application in the research of evolutionary biology and molecular phylogeny. In this study, we sequenced the whole chloroplast genome of *A. lutescens* to provide useful molecular information for the taxonomic and phylogenetic study of *Arachis*.

## Materials and methods

Fresh leaves of *A. lutescens* were collected from the Henan Academy of Agricultural Sciences, Zhengzhou, China (34.7893°N, 113.6822°E). The specimen was deposited at the Herbarium of Zhengzhou University with the voucher number Zw17 (contact: Yuhua Shi, syh@zzu.edu.cn; http://www5.zzu.edu.cn/lifesci/info/1261/3241.htm). Total genomic DNA was extracted with the Tiangen Plant Genomic DNA Kit. Genome sequences were constructed using the NovaSeq 6000 platform with a paired-end read length of 150 bp. Upon completion, more than 6.0 GB raw reads were retrieved for each sample. The complete chloroplast genome of *A. lutescens* was de novo assembled via GetOrganelle toolkit (Jin et al. 2020). The Plastid Genome Annotator (PGA) software (Qu et al. 2019) was employed to annotate the chloroplast genome, and GeSeq programs (Tillich et al. 2017) implemented in the CHLOROBOX web toolbox (https://chlorobox.mpimp-golm.mpg.de/geseq.html) were used to correct the annotation with a default setting. The complete chloroplast genome sequence of *A. hypogaea* (MW167279) was used as a reference. The circular genome map and detailed structure of the chloroplast genome were drawn using the CPGview package (Liu et al. 2023). All 13 reported chloroplast genome species, which represent different types of genomes were obtained from NCBI GenBank to reconstruct the phylogenetic tree, with *Dalbergia hupeana* Hance as an outgroup. All sequences were aligned using MAFFT software version 7.487 (Katoh and Standley 2013). And maximum-likelihood (ML) phylogenetic analyses were conducted by using RAxML v.8.2.11 (Stamatakis 2014) under GTRCAT model with 1000 bootstrap replicates. Then, whole chloroplast genomes were constructed into a ML phylogenetic tree implemented in IQtree tool (Nguyen et al. 2014), and a Bayesian inference (BI) tree using the MrBayes (Ronquist et al. 2012).

## Results

**Figure 2.**
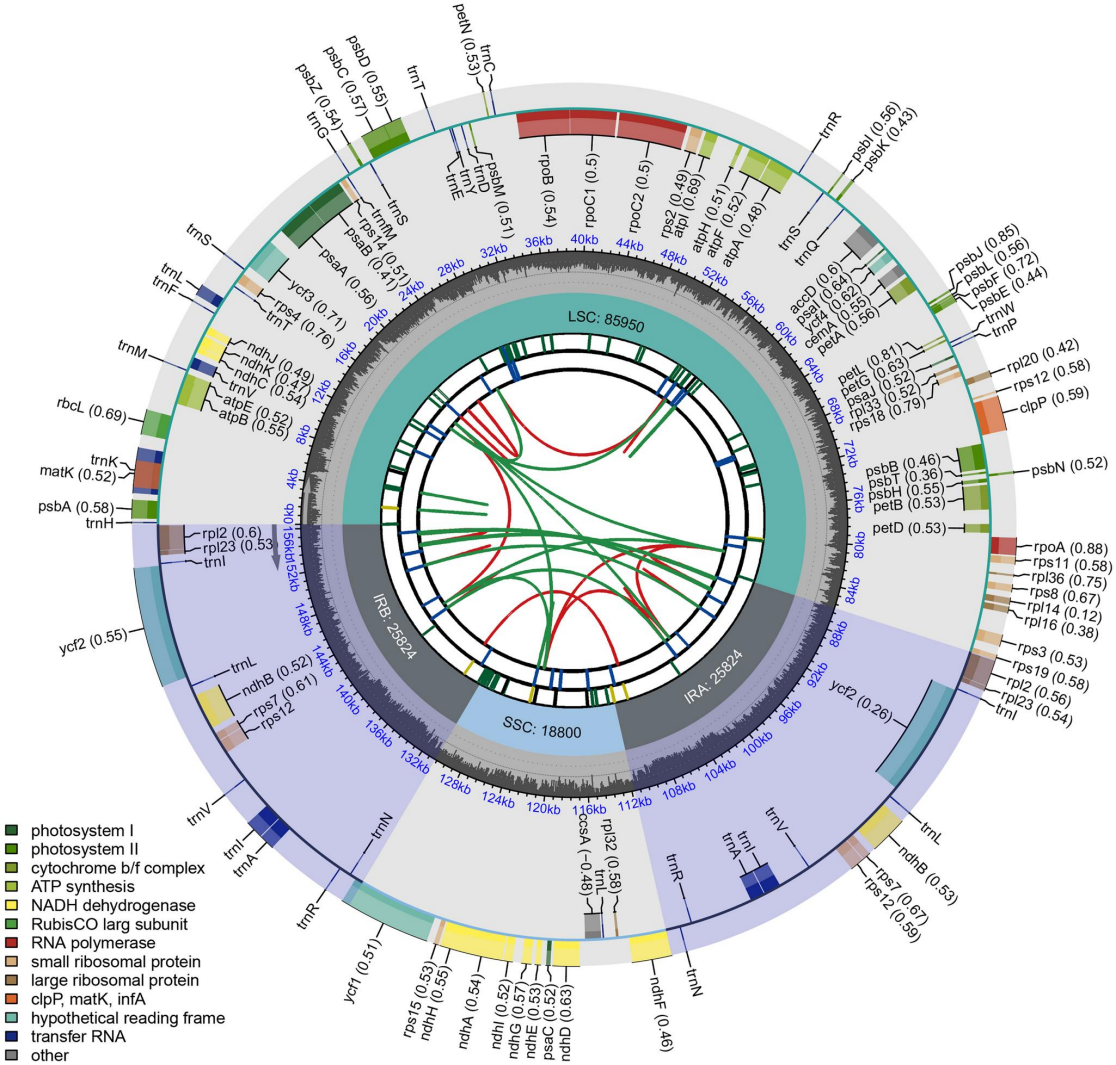
Circular map of the complete chloroplast genome of *A. lutescens* generated by CPGview. The map contains six tracks in default. From the center outward, the first track shows the dispersed repeats. The dispersed repeats consist of direct (D) and Palindromic (P) repeats, connected with red and green arcs. The second track shows the long tandem repeats as short blue bars. The third track shows the short tandem repeats or microsatellite sequences as short bars with different colors. The small single-copy (SSC), inverted repeat (IRa and IRb), and large single-copy (LSC) regions are shown on the fourth track. The GC content along the genome is plotted on the fifth track. The genes are shown on the sixth track. The optional codon usage bias is displayed in the parenthesis after the gene name. Genes are color-coded by their functional classification. The transcription directions for the inner and outer genes are clockwise and anticlockwise, respectively. The functional classification of the genes is shown in the bottom left corner.

**Figure 3.**
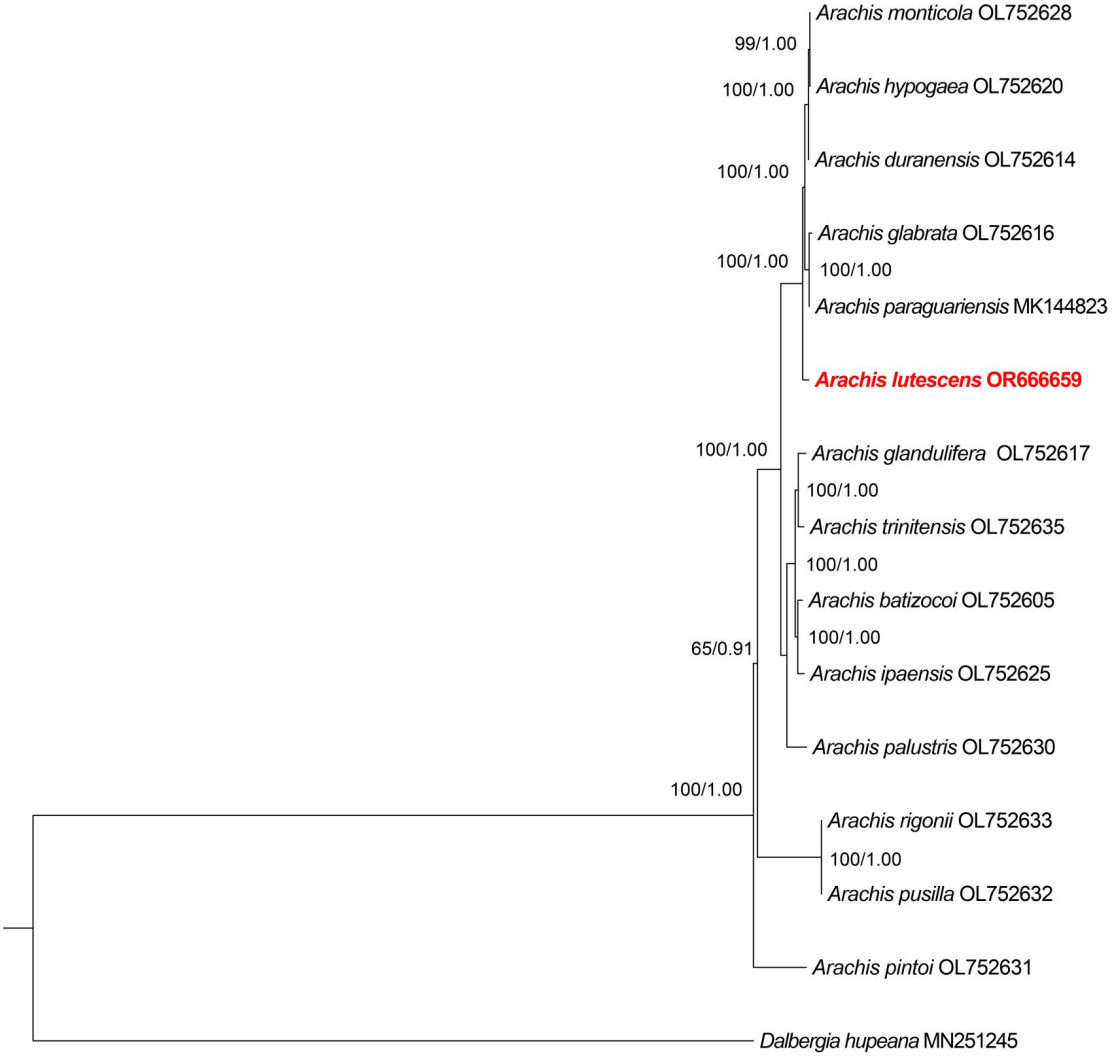
Maximum-likelihood and Bayesian inference phylogenetic tree based on the complete chloroplast genomes of 14 species of *Arachis*, with *Dalbergia hupeana* as the outgroup. The following sequences were used: *Arachis monticola* OL752628, *Arachis hypogaea* OL752620, *Arachis duranensis* OL752614, *Arachis glabrata* OL752616, *Arachis lutescens* OR666659, *Arachis glandulifera* OL752617, *Arachis trinitensis* OL752635, *Arachis ipaensis* OL752625, *Arachis batizocoi* OL752605, *Arachis palustris* OL752630, *Arachis rigonii* OL752633, *Arachis pusilla* OL752632, *Arachis pintoi* OL752631 (Tian et al. 2021), *Arachis paraguariensis* MK144823 (Wang et al. 2019), and *Dalbergia hupeana* MN251245.

The assembly result demonstrates the full-length complete chloroplast genome of *A. lutescens* is 156,398 bp in length, with a coverage depth of 1358× (Supplementary Figure 1). The chloroplast genome of *A. lutescens* with typical quadripartite structural organization (Figure 2), contains a pair of inverted repeats (IRs) of 25,824 bp separated by a large single-copy (LSC) region of 85,950 bp and a small single-copy (SSC) region of 18,800 bp. The overall GC content of chloroplast genome sequences is 36.3%, and the respective values for the LSC, SSC, and IR regions were 33.8%, 30.2%, and 42.9%. The chloroplast genome has 125 genes in total, including 81 protein-coding genes (PCGs), eight ribosomal RNA genes (rRNAs), and 36 tRNA genes (tRNAs). Among the PCGs, five PCGs have one intron (*rpoC1*, *atpF*, *rpl2*, *ndhA*, and *ndhB*), and two have two introns (*ycf3* and *clpP*) (Supplementary Figure 2). Most genes are occurred in a single copy, while 16 genes are duplicated, including four rRNAs (*rrn16*, *rrn23*, *rrn4.5*, and *rrn5*), seven tRNAs (*trnN-GUU*, *trnR-ACG*, *trnA-UGC*, *trnI-GAU*, *trnV-GAC*, *trnL-CAA*, and *trnI-CAU*), and four PCGs (rps7, ndhB, ycf2, and rpl2). We also found one trans-splicing gene *rps12* (Supplementary Figure 3). To examine the phylogenetic position of *A. lutescens*, a phylogenetic analysis was performed based on the complete chloroplast genome sequences of *Arachis* species, which represent different genome types. The phylogenetic trees generated with ML and BI methods showed the same topology with high bootstrap values (Figure 3). *A. lutescens* are clustered together with *A. monticola*, *A. hypogaea*, *A. duranensis*, *A. paraguariensis*, and *A. glandulifera*, in a well-supported clade. These findings provide a foundation for further studies on the evolutionary relationships of *A. lutescens*.

## Discussion and conclusions

In this study, the whole chloroplast genome of *A. lutescens* was assembled and annotated for the first time. The genomic structure of *A. lutescens* consists of a pair of IRs, an SSC region, and an LSC region similar to that in the majority of other angiosperms. The genome size and gene content of *A. lutescens* are not significantly different from those of most chloroplast genomes or plastomes in the genus *Arachis* (Prabhudas et al. 2016; Yin et al. 2017; Wang et al. 2018, 2019; Tian et al. 2021). Furthermore, the phylogenetic analysis based on ML and BI methods showed the same topology. This phylogenetic result is similar to the phylogenetic analysis of whole or PCGs chloroplast DNA sequences (Yin et al. 2017; Wang et al. 2019; Tian et al. 2021). In addition, *A. lutescens* was included in the sect. *Extranervosae*, which shows EX genome type, while other species have the AABB (*A. monticola*, *A. hypogaea*), AA (*A. duranensis*), EE (*A. paraguariensis*), and DD (*A. glandulifera*) genome types. In summary, our results provide a reference for the genetic evaluation of *A. lutescens*. The genetic resource information generated in this study will be valuable for further investigations into the biology and evolutionary history of peanut and its wild species.

## Supplementary Material

Supplemental Material

## Data Availability

The genome sequence data that support the findings of this study are openly available in GenBank of NCBI (https://www.ncbi.nlm.nih.gov/ OR666659) under accession no. OR666659. The associated BioProject, SRA, and Bio-Sample numbers are, PRJNA1028390, SRR26391381, and SAMN37824455, respectively.
