# Supplementary material for: The complete chloroplast genome of Arachis lutescens Krapov. & Rigoni (Fabaceae)
Source: Mitochondrial DNA B Resour. 2024 May 31;9(6):687–91. doi: 10.1080/23802359.2024.2353230 (PMC11146263; doi:10.1080/23802359.2024.2353230)
Supplement: Supplemental Material [file TMDN_A_2353230_SM5586.docx]

Figure captions

Supplementary Figure 1. Overall coverage depth of the chloroplast genome assembly of *Arachis lutescens*.

Supplementary Figure 2. Schematic map of the cis-splicing genes in the *Arachis lutescens* chloroplast genome.

Supplementary Figure 3. Schematic map of the trans-splicing gene rps12 in the *Arachis lutescens* chloroplast genome.

Supplementary Figure 1. Overall coverage depth of the chloroplast genome assembly of *Arachis lutescens*.


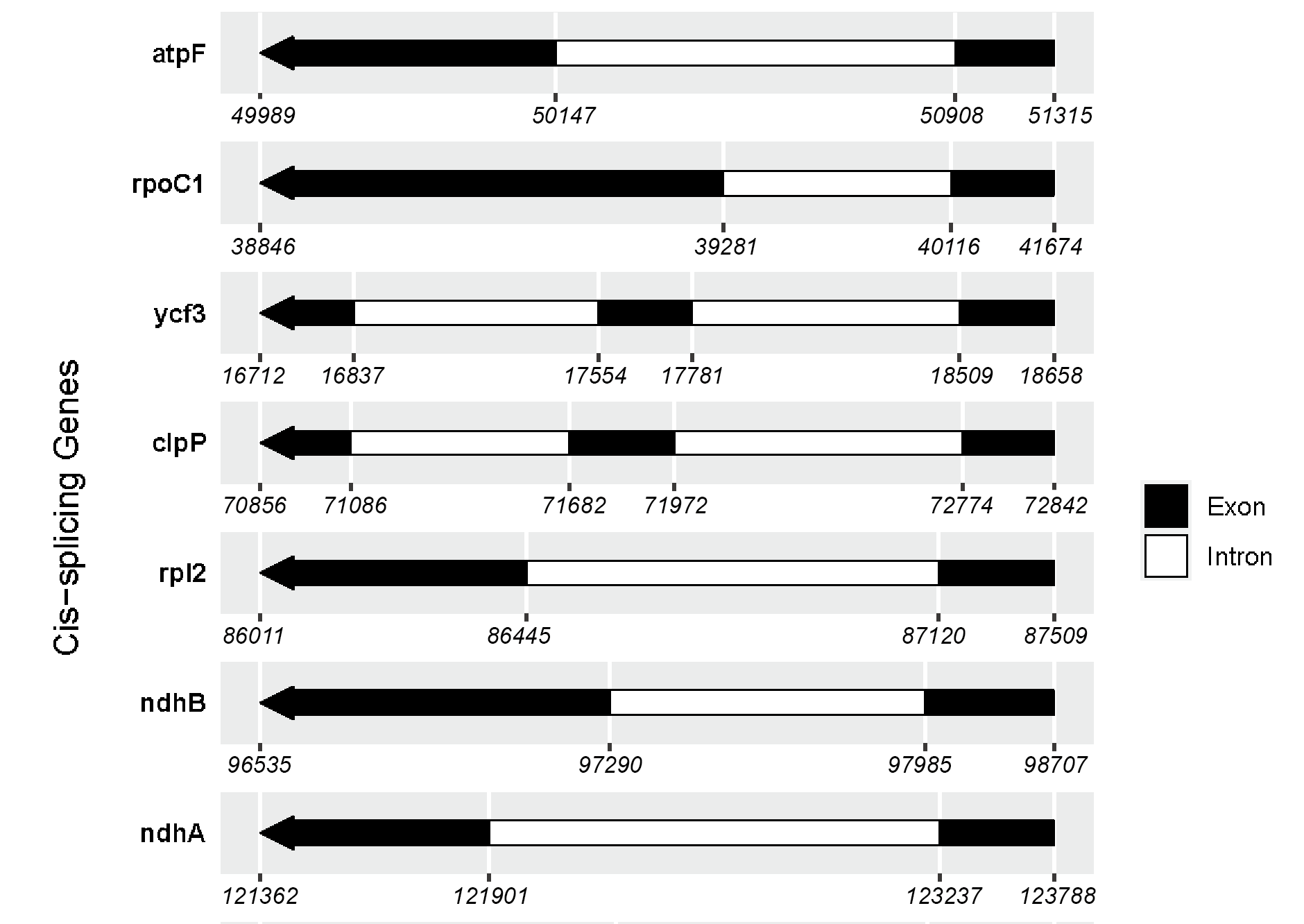


Supplementary Figure 2. Schematic map of the cis-splicing genes in the *Arachis lutescens* chloroplast genome.


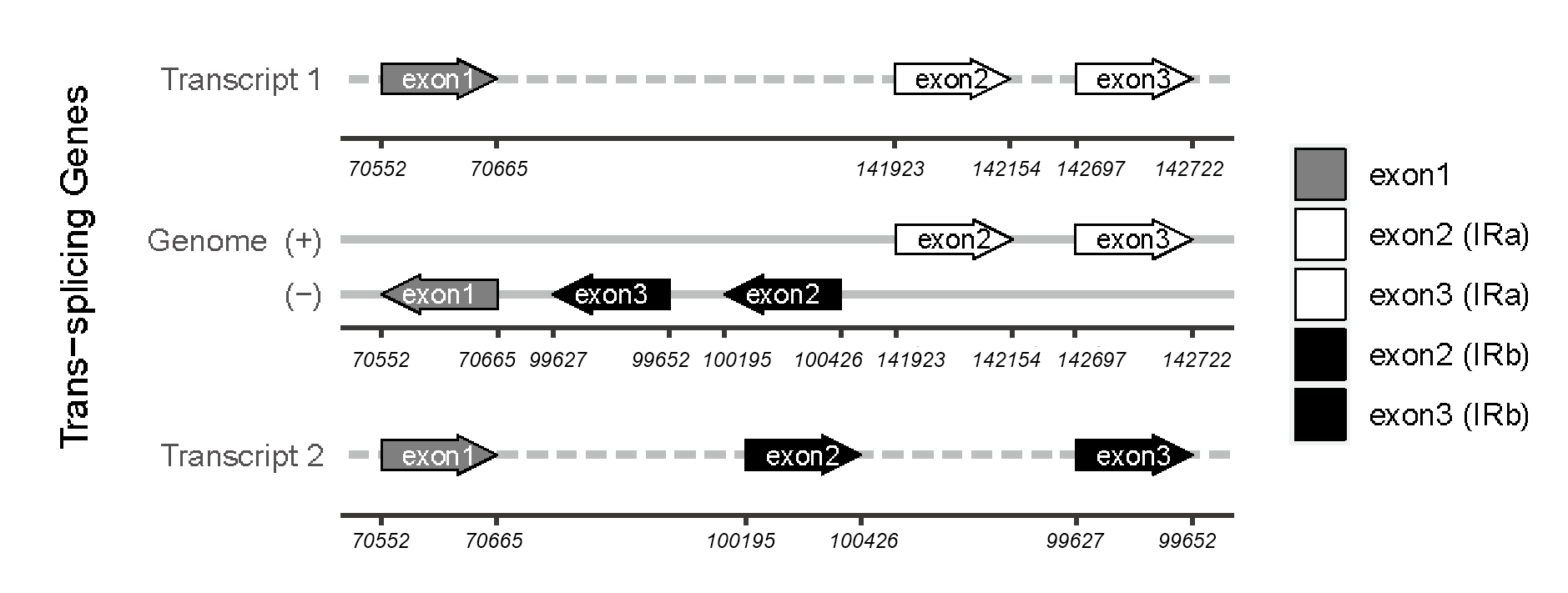


Supplementary Figure 3. Schematic map of the trans-splicing genes in the *Arachis lutescens* chloroplast genome.
